# Characteristics of the complete mitochondrial genome of the monotypic genus *Arctictis* (Family: Viverridae) and its phylogenetic implications

**DOI:** 10.7717/peerj.8033

**Published:** 2019-11-25

**Authors:** Siuli Mitra, Vaishnavi Kunteepuram, Klaus-Peter Koepfli, Neha Mehra, Wajeeda Tabasum, Ara Sreenivas, Ajay Gaur

**Affiliations:** 1 Laboratory for Conservation of Endangered Species (LaCONES), CSIR-Centre for Cellular and Molecular Biology, Hyderabad, Telangana, India; 2 Center for Species Survival, Smithsonian Conservation Biology Institute, Washington, D.C., USA

**Keywords:** Mitochondrial DNA, Time calibrated phylogeny, Arctictis binturong albifrons, Viverridae

## Abstract

The binturong (*Arctictis binturong*) is classified as a member of the subfamily Paradoxurinae within the family Viverridae (Carnivora: Mammalia) and comprises nine subspecies spread across Southern and Southeast Asia. Here, we describe the complete mitochondrial genome of the Indian subspecies *A. b. albifrons* using next-generation sequencing methods. The total length of the *A. b. albifrons* mitogenome was 16,642 bp. Phylogenetic analyses based on 13 mitochondrial protein-coding genes placed the binturong as a sister taxon to *Paguma larvata* within the Paradoxurinae and supported the clustering of *Genettinae* and *Viverrinae* and the monophyly of Viverridae and six other families of feliforms, consistent with previous studies. Divergence time estimates suggest that the Viverridae diversified during the Miocene (22.62 Mya: 95% CI [20.78–24.54] Mya) and that *Arctictis* and *Paguma* split 12.57 Mya (95% CI [8.66–15.67] Mya). Further molecular studies are required to test the distinctiveness and diversity of the nine putative subspecies of binturong.

## Introduction

*Arctictis binturong* (Raffles, 1822), commonly called binturong or bearcat, is the largest known member of the Viverridae (Carnivora: Mammalia) and is characterized by coarse, black fur and a prehensile tail (Pocock, 1933). In forest ecosystems of Southeast Asia, the frugivorous binturong has co-evolved with fig trees to form a keystone relationship, wherein the animal facilitates and propagates seed germination while the fig tree provides a stable dietary source (Kinnaird & O’Brien, 2007). Binturongs are presently being poached for their meat, traditional medicines and the pet trade, and alongside habitat destruction, these factors have contributed to decreasing the numbers of binturong to a few geographical pockets across the species’ former range (Willcox et al., 2016). As a result of these increasing pressures, the binturong is listed as ‘Vulnerable’ on the IUCN Red List of Threatened Species (Willcox et al., 2016).

Nine subspecies of *A. binturong* have been described primarily on the basis of region-specific variations in fur color (Pocock, 1933; Cosson et al., 2006). In addition, shared morphological similarities with other viverrids like perineal scent glands and syndactyly of the third and fourth digits of the hind foot, along with the unique features of completely naked soles of the hind feet and a prehensile tail, have helped determine the phylogenetic position of binturong within the viverrid subfamily Paradoxurinae (Pocock, 1933; Gregory & Hellman, 1939; Veron, 2007). More recent molecular phylogenetic studies indicate that this subfamily also includes *Paguma*, *Arctogalidia* and *Macrogalidia* (Albert, 2001; Cosson et al., 2006; Patou et al., 2008; Nyakatura & Bininda-Emonds, 2012; Zhou, Wang & Ma, 2017). However, the genetic structure of the binturong has not been studied in detail (but see Cosson et al., 2006), which will be essential to validate the evolutionary and conservation genetic implications of the existence of nine geographically and morphologically disparate subspecies.

Mohd Salleh et al. (2017) reported the first mitochondrial genome of a binturong as part of a larger study to generate an expanded reference mitogenome dataset of mammals from Southeast Asia that could be applied for monitoring mammalian biodiversity using environmental DNA approaches. However, the sequence reported in that study came from an animal at the Tier Park Berlin Zoo of unknown provenance. Moreover, the sequence contains many missing nucleotides (Ns) and is therefore incomplete. To rectify this, we generated a complete (gapless) mitochondrial genome sequence from a wild-caught binturong of known provenance belonging to the Indian subspecies, *Arctictis binturong albifrons*. The aims of our study were to: (a) characterize the *Arctictis* mitogenome in comparison with other viverrids and feliforms, and (b) provide the first molecular phylogenetic and divergence dating analysis of the *Arctictis* in the context of Viverridae and other feliform families based on whole mitochondrial genomes.

## Materials and Methods

### Sampling, Extraction and PCR amplification

A blood sample of an individual identified as *A. b. albifrons* was collected and forwarded by the Veterinary Assistant Surgeon of the Sepahijala Zoo, Tripura (Vide Letter No. F5(D)VD/Sep/Sl No. 100-102, dated 19/07/2008) for DNA analysis, and deposited in the Genome Bank at the Laboratory for Conservation of Endangered Species, CCMB, Hyderabad, India. Genomic DNA was isolated from the blood sample by the phenol-chloroform-isoamyl alcohol method (Sambrook, Fritsch & Maniatis, 1989) and the DNA integrity was checked electrophoretically in a 0.8% agarose gel. The mitochondrial genome was amplified by long range PCR using the TaKaRa LA PCR kit v2 (Takara Bio Inc, USA) following the manufacturer’s recommendations. Three PCR products of 4.1 kbp, 8.8 kbp and 4.5 kbp were generated using three sets of primers (Table S1).

### Genome sequencing, assembly and annotation

Next-generation sequencing libraries were constructed in three steps: enzymatic shearing, adapter ligation and fragment size selection. PCR product quality was first assessed using the Qubit^®^ 2.0 Fluorometer (Life Technologies, USA). Amplified products were then subjected to enzymatic fragmentation to an average of 550 bp fragments using the Covaris M220 system (Covaris, USA). Libraries were prepared with the TruSeq DNA PCR-Free Sample Preparation Kit (Illumina, USA) following the manufacturer’s recommendations. The High Sensitivity DNA Analysis Kit (Agilent Technologies, USA) was used for quantification and size estimation of the libraries generated on a 2100 Bioanalyzer (Agilent Technologies, USA). The libraries were standardized to 1.5pM and sequenced using the NextSeq 500 sequencer (Illumina, USA). The paired end reads generated on Illumina were used for reference assembly. Raw sequences were extracted in the FASTQ format and checked for quality using the CLC Genomics Workbench v 9.0 software (https://www.qiagenbioinformatics.com/). Raw sequences filtering was performed by trimming adaptors. Nucleotides and sequence reads showing ambiguity or low quality scores (<Q20) were excluded from further analysis. High quality data obtained after filtering was assembled and annotated using CLC Genomic Work bench v 9.0. Mauve version 2.4.0 (Darling et al., 2004) was used for the comparative reference assembly. The binturong mitogenome sequence was analyzed using CLC Genomic Workbench v 9.0. to identify the mitochondrial gene locations, their order, and start and end points.

### Genome analysis

The circular map of mitogenome was created using Geneious R 10.1 (Kearse et al., 2012). Sequences of the 13 protein-coding genes were translated into amino acid sequences using ORF finder (https://www.ncbi.nlm.nih.gov/orffinder/) to verify orthology with other feliform taxa and exclude the potential presence of NUMTs (nuclear-mitochondrial paralogues). The control region was extracted and scanned for the presence of palindromes and other repeats using the EMBOSS and REPFIND tools (Rice, Longden & Bleasby, 2000; Betley et al., 2002). We evaluated the base composition of the binturong mitogenome and those of 11 other feliform species using Geneious R 10.1 (Table S2).

### Phylogenetic analysis and estimation of divergence times

The phylogenetic relationships of the binturong within Viverridae and Feliformia were reconstructed by aligning its 13 protein-coding gene sequences with those of 22 feliform species comprising five viverrids, 11 felids, two hyaenids, and one species each from Nandiniidae, Herpestidae, Eupleridae and Prionodontidae (Table S2). The *Cuon alpinus* mitogenome (Canidae, NCBI Accession No. NC_013445.1) was also included as outgroup to root the feliform tree. Sequences were aligned using MEGA 6.06 with the default parameters of CLUSTALW (Thompson, Higgins & Gibson, 1994) and then concatenated, resulting in an 11,313 bp alignment. Maximum likelihood phylogenetic analysis was conducted using raxmlGUI v1.3 (Silvestro & Michalak, 2012). Support for different nodes was estimated using 1,000 bootstrap replicates (ML + bootstrap option) under the GTR+I+G model, as estimated with jModeltest 2.1.5 (Darriba et al., 2012) and Bayesian Inference (BI) method using Mr. Bayes v.3.2.5.

We jointly estimated the phylogeny and divergence times between species within a Bayesian inference framework using the program BEAST v1.7.5 (Drummond & Rambaut, 2007). The 13 protein-coding genes were partitioned and the appropriate model of sequence evolution was determined by the BIC criteria in jModeltest 2.1.5 (Darriba et al., 2012). Substitution rates were estimated under a relaxed uncorrelated clock model [14] for 1 ×10^8^ million generations, sampling every 1,000th generation to allow for adequate mixing. Base frequencies were set to “All equal” and the number of gamma categories was set to 4. Four fossil priors with a uniform probability distribution were used for calibration: (a) Minimum and maximum ages of split between Caniformia and Feliformia were set at 43 Mya and 63.8 Mya, respectively (Benton & Donoghue, 2007) (b) Minimum age of origin of *Viverridae* was set at 23 Mya (Hunt, 1991; Hunt Jr, 1996) (c) Minimum age of the split between *Crocuta* and *Hyaena* was set 9.5 Mya (Wozencraft, 2005); and (d) Minimum and maximum ages of origin of Felidae were set at 5.3 Mya and 23 Mya (McKenna & Bell, 1997). The first 25% of MCMC iterations were removed from the posterior sample as burn-in. Convergence was monitored in Tracer (Ver. 1.4) to determine if effective sampling sizes (ESS) were adequate (>200). Trees were summarized with Tree Annotator and represented as the maximum clade credibility tree.

## Results and Discussion

### Binturong mitogenome assembly and annotation

**Figure 1:**
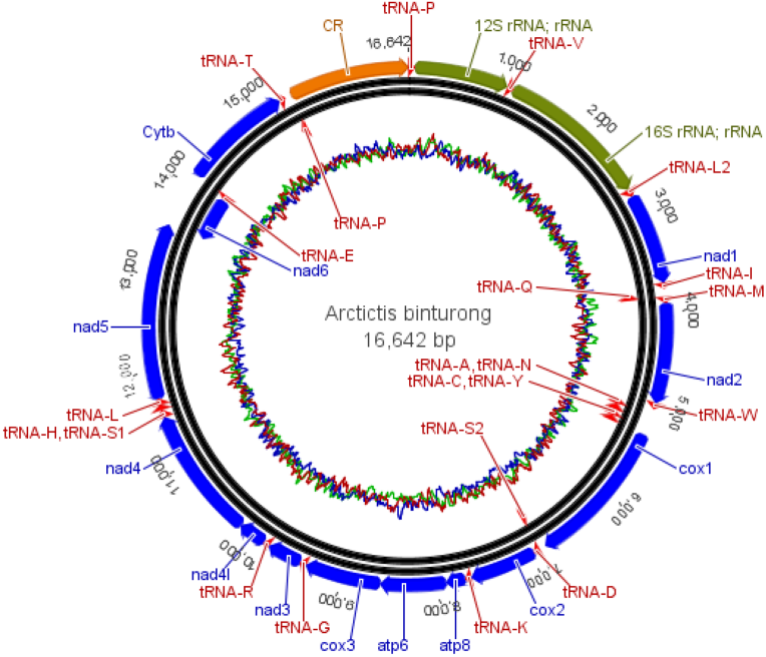
The complete mitogenome organization of *A. binturong albifrons*. Transfer RNAs (tRNA) are labelled with their corresponding amino acid and are shown in pink; COI, COII and COIII refer to subunits of cytochrome c oxidase; Cytb referes to cytochrome b; 12S rRNA and 16s rRNA refer to ribosomal RNAs; ND1-ND6 refer to components of NADH dehydrogenase; ATPase 6 and ATPase 8 refer to classes ATPsynthase. Blue color: coding genes, green color: rRNAs and yellow color: control region.

A total of 2,726,370 high-quality reads were obtained after filtering and trimming sequences. The assembly of the binturong mitogenome resulted in a total of 162 contigs with an N50 length of 1074 bp and the length of contigs ranging from 501 bp to 16,752 bp. After trimming ends, the *A. b. albifrons* mitogenome was 16,642 bp in length (Fig. 1). The assembly size of the binturong mitogenome reported by Mohd Salleh et al. (2017) is 17,067 bp, 425 bp longer than our assembly. However, this increased length is due to the presence of inserted Ns (missing nucleotides) within the sequence. The *A. b. albifrons* mitogenome is generally shorter when compared with those of nine other feliform species (Table S2). The *A. b. albifrons* mitogenome sequence was deposited in NCBI GenBank (accession number KX449332).

Annotation yielded a total of 37 genes: 13 protein-coding genes (PCGs), 22 tRNAs, 2 rRNAs and the non-coding control (D-loop) region (Table 1). No NUMTs were detected among the 13 PCGs when they were translated into amino acids (no insertion, deletions, frame shift mutations or premature stop codons). Analysis of base composition showed a bias towards higher adenine and thymine content in the binturong mitogenome (Table 2), amounting to 64.63% and individual base proportions amounting to 32.80% A, 31.83% T, 16.46% G and 18.91% C. This pattern is consistent with the base composition of twelve other feliform species, with AT content ranging from 60.28% in *Hyaena* to 64.64% in *P. larvata* while a higher average bias was maintained within Viverridae (65.02%). The control region was 1,234 bp long, located from 15,408–16,642 bp in the binturong mitogenome (Fig. 1). The region included five mononucleotide T stretches, two of (T)_4_ and three of (T)_5_. A 12 bp long palindrome sequence (5′-TATCTATAGATA- 3′) was located between nucleotide positions 890-901 in the alignment of the control region sequences of the binturong and 11 other feliform species.

**Table 1:** Characteristic features of the binturong mitogenome (H denotes Heavy Strand; L denotes Light Strand).

**Gene**	**Strand**	**Start**	**End**	**Size (bp)**	**Start codon**	**Stop codon**	**Base composition (%)**		**AT SKEW**	**GC SKEW**
							**(A+T)%**	**(G+C)%**		
*tRNAF*^*Phe*^	H	1	69	68	–	–	60	39	0.26	−0.02
*12S rRNA*	H	70	1,034	964	–	–	59	39	0.25	−0.17
*tRNAV*^*Tyr*^	H	1,035	1,103	68	–	–	59	39	0.22	−0.28
*16S rRNA*	H	1,104	2,678	1,574	–	–	62	37	0.19	−0.08
*tRNAL2*	H	2,679	2,754	75	–	–	58	40	0	−0.1
*nad1*	H	2,755	3,697	942	ATG	AGA	60	38	0	−0.4
*tRNAI*^*Asp*^	H	3,698	3,767	69	–	–	72	26	0.08	0.15
*tRNAQ*^*Leu*^	L	3,768	3,841	73	–	–	66	32	0.09	−0.4
*tRNAM*^*His*^	H	3,842	3,911	69	–	–	55	42	0.01	−0.25
*nad2*	H	3,912	4,944	1,032	ATT	AGA	65	33	0.13	−0.51
*tRNAW*^*Ser*^	H	4,945	5,015	70	–	–	57	41	0.12	−0.17
*tRNAA*^*Cys*^	L	5,016	5,085	69	–	–	64	34	0.15	−0.35
*tRNAN*^*Val*^	L	5,086	5,159	73	–	–	60	39	0	−0.2
*tRNAC*^*Ala*^	L	5,160	5,228	68	–	–	56	42	−0.07	−0.19
*tRNAY*^*Val*^	L	5,229	5,297	68	–	–	60	74	0	0.4
*cox1*	H	5,298	6,831	1,533	ATG	TAA	62	36	−0.1	−0.11
*tRNAS2*	L	6,832	6,901	69	–	–	63	35	0.17	−0.2
*tRNAD*^*Val*^	H	6,902	6,971	69	–	–	70	28	−0.05	0.2
*cox2*	H	6,972	7,653	681	ATG	TAA	64	34	0.03	−0.2
*tRNAK*^*Phe*^	H	7,654	7,722	68	–	–	72	26	0.08	0
*atp8*	H	7,723	7,921	198	ATG	TAA	71	27	0.12	−0.5
*atp6*	H	7,922	8,597	675	ATG	TAA	63	34	−0.01	−0.5
*cox3*	H	8,598	9,381	783	ATG	TAG	61	37	−0.04	−0.4
*tRNAG*^*Ser*^	H	9,382	9,452	70	–	–	65	33	0.04	−0.09
*nad3*	H	9,453	9,798	345	ATA	TA	64	34	0	−0.35
*tRNAR*^*Ser*^	H	9,799	9,868	69	–	–	77	21	0.14	−0.04
*nad4l*	H	9,869	10,163	294	ATG	TAA	64	33	−0.09	−0.33
*nad4*	H	10,164	11,532	1,368	ATG	TA	64	34	0.03	−0.41
*tRNAH*^*Val*^	H	11,533	11,602	69	–	–	76	22	0.02	0s
*tRNAS1*^*Ala*^	H	11,603	11,662	59	–	–	65	33	0.07	−0.09
*tRNAL*^*Ser*^	H	11,663	11,733	70	–	–	68	31	0.2	0.09
*nad5*	H	11,734	13,540	1,806	ATT	TAA	65	34	0.01	−0.4
*nad6*	L	13,541	14,063	522	ATG	TAA	62	35	0.32	−0.4
*tRNAE*^*Phe*^	L	14,064	14,133	69	–	–	69	29	0.13	−0.24
*Cob*	H	14,134	15,268	1,134	ATG	AGA	60	38	0	−0.31
*tRNAT*^*Cys*^	H	15,269	15,340	71	–	–	63	34	0.04	−0.11
*tRNAP*^*Trp*^	L	15,341	15,407	66	–	–	55	43	0.23	−0.39
Control region	H	15,408	16,642	1,234	–	–	61	37	0.01	−0.29

**Table 2:** Genome length, base composition, (A+T) percentage and AT and GC skewness in mitogenomes of binturong and nine other feliforms.

**Species**	**Size (bp)**	**A%**	**G%**	**T%**	**C%**	**(A+T)%**	**AT skew**	**GC skew**
**WHOLE MITOCHONDRIAL GENOME**								
*A. binturong*	16,642	32.75	16.45	31.81	18.99	63.64	0.07	−0.19
*A. binturong*	17,067	33.1	12.9	29.2	23.5	62.3	0.06	−0.29
*C. bennettii*	15,785	34.2	12.3	31.0	22.6	65.2	0.04	−0.29
*P. larvata*	16,710	33.43	16.11	30.97	19.49	64.64	0.04	−0.09
*G. servalina*	16,938	32.90	16.72	30.08	20.30	62.98	0.04	−0.09
*V. indica*	16,583	32.99	16.41	30.66	19.94	63.65	0.04	−0.09
*H. javanicus*	16,758	32.38	17.00	29.96	20.66	62.34	0.04	−0.09
*M. decemlineata*	16,905	31.74	17.81	28.76	21.7	60.50	0.05	−0.09
*H. hyaena*	17,112	31.58	17.93	28.62	21.87	60.28	0.05	−0.09
*F. catus*	17,009	32.82	16.92	29.56	20.70	62.38	0.05	−0.09
*P.pardicolor*	16,718	32.50	16.77	30.76	19.98	63.25	0.03	−0.08
*N. binotata*	17,087	33.12	16.51	29.54	20.84	62.65	0.06	−0.11
**PROTEIN CODING GENES (PCGs)**								
*A. binturong*^a^	11,313	30.99	12.71	33.15	23.15	63.46	0.03	−0.37
*A. binturong*	11,444	31.8	12.4	31.5	24.1	63.3	0.004	−0.31
*C. bennettii*	11,332	32	11.9	33.3	22.6	65.3	−0.01	−0.31
*P. larvata*	11,316	31.89	12.48	32.41	23.22	64.30	−0.01	−0.30
*G. servalina*	11,410	31.02	13.24	30.41	25.33	61.44	0.01	−0.32
*V. indica*	11,410	31.45	12.86	31.30	24.39	62.75	0.00	−0.31
*H. javanicus*	11,301	31.12	13.60	29.44	25.84	60.56	0.03	−0.31
*M. decemlineata*	11,295	29.83	14.71	27.33	28.13	57.16	0.04	−0.31
*H. hyaena*	11,310	30.22	14.50	27.54	27.74	57.77	0.05	−0.31
*F. catus*	11,292	30.94	13.74	28.79	26.52	59.74	0.03	−0.32
*P. pardicolor*	11,286	30.61	13.66	30.79	24.94	61.40	0.00	−0.29
*N. binotata*	11,303	31.74	12.64	28.23	27.39	59.97	0.06	−0.37
**tRNA**								
*A. binturong*^a^	1,519	33.45	18.63	31.77	16.14	64.09	0.08	0.07
*A. binturong*	1,582	33.5	18.83	30.7	16.87	64.28	0.04	0.05
*C. bennettii*	1,516	33.9	18.46	31.26	16.2	65.16	0.04	0.06
*P. larvata*	1,517	34.14	18.20	31.09	16.58	65.22	0.05	0.05
*G. servalina*	1,512	33.71	18.79	30.63	16.86	64.35	0.05	0.05
*V. indica*	1,515	33.61	18.50	31.06	16.82	64.67	0.04	0.05
*H. javanicus*	1,514	32.77	19.07	31.09	17.07	63.86	0.03	0.05
*M. decemlineata*	1,512	32.41	19.75	30.38	17.46	62.79	0.03	0.06
*H. hyaena*	1,512	31.91	20.07	30.07	17.95	61.98	0.03	0.06
*F. catus*	1,515	33.62	18.80	30.77	16.81	64.39	0.04	0.05
*P. pardicolor*	1,514	33.39	18.54	31.55	16.51	64.94	0.03	0.05
*N. binotata*	1,513	33.60	18.83	31.17	16.40	64.77	0.04	0.07
**rRNA**								
*A. binturong*^a^	2,538	37.31	16.97	23.93	21.80	60.5	0.22	−0.13
*A.binturong*	2,537	37.24	18.8	24	21.75	61.2	0.21	−0.12
*C.bennettii*	2,537	37.24	17.06	25.10	20.57	62.34	0.19	−0.09
*P. larvata*	2,537	36.68	17.39	23.73	22.19	60.41	0.21	−0.12
*G.servalina*	2,532	36.50	17.63	23.84	22.03	60.34	0.21	−0.11
*V.indica*	2,530	37.05	17.25	23.84	21.86	60.89	0.22	−0.12
*H. javanicus*	2,534	36.82	17.62	22.09	23.47	58.91	0.25	−0.14
*M. decemlineata*	2,535	36.46	17.96	21.95	23.63	58.41	0.25	−0.14
*H. hyaena*	2,533	35.96	18.33	22.04	23.67	58.00	0.24	−0.13
*F. catus*	2,538	36.52	18.05	22.86	22.57	59.38	0.23	−0.11
*P. pardicolor*	2,544	35.35	18.22	24.21	22.22	59.55	0.19	−0.10
*N. binotata*	2,538	37.35	17.13	21.86	23.66	59.21	0.26	−0.16
**CONTROL REGION**								
*A. binturong*^a^	1,191	30.98	15.95	31.23	21.83	62.22	0.00	−0.16
*A.binturong*	1,304	37.96	8.81	26.84	26.38	64.8	0.17	−0.49
*C.bennettii*	1,292	33.9	9.90	32.26	23.14	66.16	0.02	−0.40
*P. larvata*	1,260	31.27	14.92	24.29	29.52	55.56	0.13	−0.33
*G.servalina*	1,497	32.33	14.43	25.92	27.32	58.25	0.11	−0.31
*V.indica*	1,135	31.28	14.80	27.05	26.87	58.33	0.07	−0.29
*H. javanicus*	1,318	31.26	14.57	27.54	26.63	58.80	0.06	−0.29
*M. decemlineata*	1,423	32.19	15.04	25.37	27.41	60.5	0.12	−0.29
*H. hyaena*	1,673	33.17	14.88	23.79	28.15	56.96	0.16	−0.31
*F. catus*	1,560	32.12	14.74	26.35	26.79	58.46	0.10	−0.29
*P. pardicolor*	1,271	31.63	15.18	25.96	27.22	57.59	0.03	−0.08
*N. binotata*	1,638	31.93	14.53	25.89	27.66	57.81	0.10	−0.31

**Notes.**

a Sequence generated in this study.

### Phylogenetic relationships and divergence time estimates

Maximum likelihood and Bayesian inference analyses (Fig. S1) of the concatenated sequences of the 13 PCGs (11,313 bp) support the monophyly of Viverridae among feliforms, the monophyly of Paradoxurinae among viverrids, the clustering of Paradoxurinae and Hemigalinae, as found by Veron et al. (2017), and the clustering of Genettinae with Viverrinae as proposed by Veron (2007) and Eizirik et al. (2010). The monophyly of Viverridae was strongly supported in both the analyses, with a Bayesian Posterior Probability (bpP) of 1 and a bootstrap support (BS) of 100%. Viverrid monophyly was also observed by Patou et al. (2008) and finds morphological support as the species therein share a characteristic union of the third and fourth digits in the hind foot and a hypocarnivorous dentition (Patou et al., 2008; Veron, 2007). Within Viverridae, Paradoxurinae (*A. binturong* and *P. larvata*) and Paradoxurinae + Hemigalinae (*C. bennetti*) were both monophyletic, each with bpP of 1 and BS = 100%. Finally, Genettinae and Viverrinae were found to have a sister relationship with maximum bpP support and BS = 94%. Inferred relationships among other feliform familes were well supported and congruent with previous studies (Veron & Heard, 2000; Gaubert & Veron, 2003; Yoder et al., 2003; Flynn et al., 2005; Koepfli et al., 2006; Eizirik et al., 2010).

**Figure 2:**
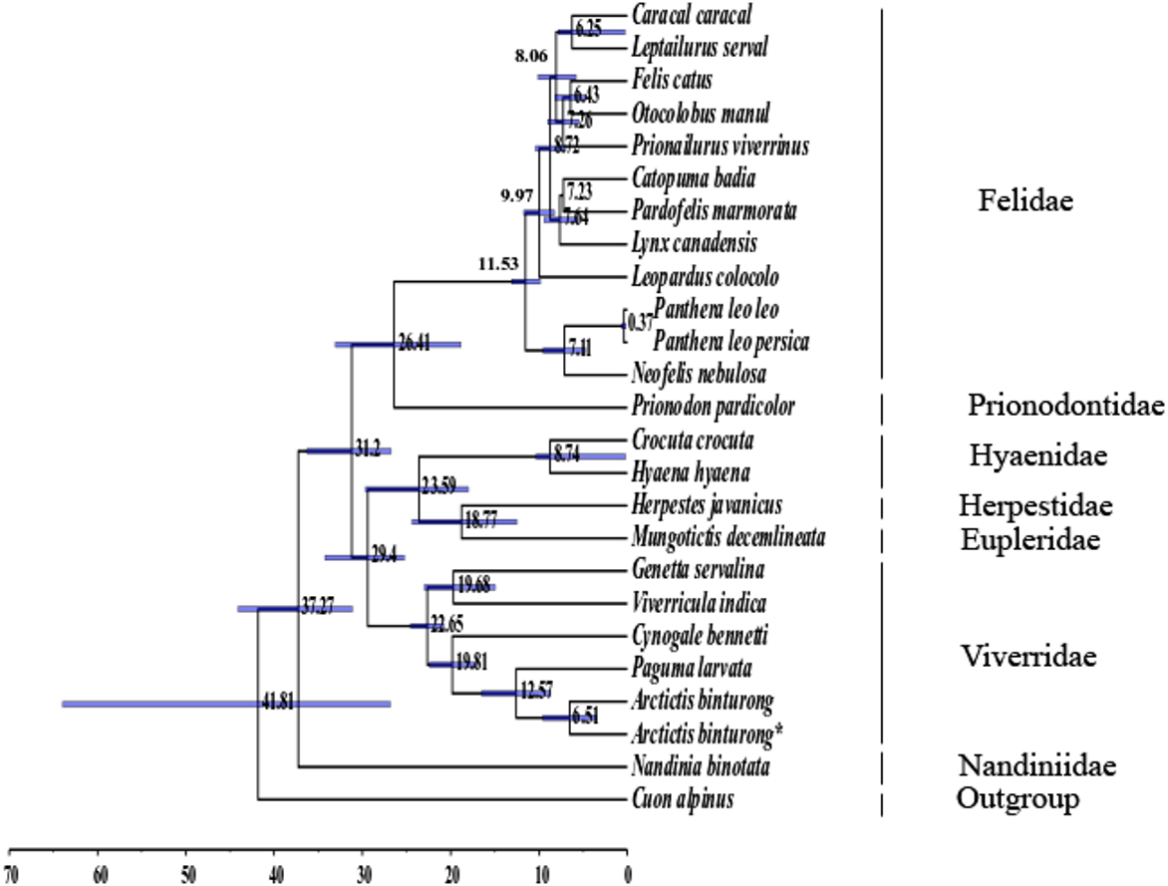
Mean divergence date estimates among 24 feliform species. The 24 feliform species (listed in Table S2 ) includes five from Viverridae showing divergence of Viverridae and sub-families Paradoxurinae (*Arctictis binturong* and *Paguma larvata*), Genettinae (*Genetta servalina*), Hemigalinae (*Cynogale bennetti*), and Viverrinae (*Viverricula indica*). Blue bars spanning nodes show 95% highest posterior density (HPD) for divergence times. Timescale in millions of years ago (Mya) is shown at the bottom. The Asiatic dhole (*Cuon alpinus*) was used as the outgroup to root the feliform tree.

Divergence time estimation (Fig. 2) showed that Viverridae began diversifying around 22.65 Mya (95% Credibility Interval (CI) [20.78 Mya –24.54 Mya]), close to the dates estimated in earlier studies (Gaubert & Veron, 2003; Koepfli et al., 2006; Patou et al., 2008; Zhou, Wang & Ma, 2017) but more recent than that suggested in other studies (Gaubert & Cordeiro Estrela, 2006; Eizirik et al., 2010). The estimates obtained from this study and most others agree with the temporal position of the earliest evidence for Viverridae, represented by the late Oligocene-early Miocene fossil *Herpestides*, which has been dated at 23 Mya (Johnson et al., 2006; Morlo, Miller & El-Barkooky, 2007). The split between *Arctictis* and *Paguma* in Paradoxurinae was estimated at 12.57 Mya (95% CI [8.66 Mya –16.51 Mya]), which agrees with the date of origin of Paradoxurinae suggested to be during the Miocene (Gaubert & Cordeiro Estrela, 2006; Veron, 2007). Divergence times estimated for the origin of Feliformia (41.80 Mya, CI [26.82–63.98]) and the feliform families are consistent with those reported in previous studies (Gaubert & Cordeiro Estrela, 2006; Koepfli et al., 2006; Eizirik et al., 2010; Zhou, Wang & Ma, 2017).

## Conclusions

We have reported the first complete (gapless) mitogenome of the binturong, representing the Indian subspecies *Arctictis binturong albifrons*. The binturong mitogenome, along with the one previously reported by (Mohd Salleh et al., 2017), provides a starting point for further testing the distinctiveness and diversity of the nine putative subspecies of binturong and thereby provide critical information for designing conservation management plans for this vulnerable species.

##  Supplemental Information

10.7717/peerj.8033/supp-1 Table S1 PCR Primer information used in this study

10.7717/peerj.8033/supp-2 Table S2 Mitogenomes used for comparative characterization and phylogenetic analysis, name of species, family and accession numbers The accession no. (KY117560) supplied for Binturong in Mohd Salleh et al. (2017) is in fact the accession for “Muntiacus muntjak mitochondrion, partial genome”. The Binturong sequence was obtained by contacting the authors directly.

10.7717/peerj.8033/supp-3 Figure S1 Phylogenetic relationships among mitogenomes of Feliformia, reconstructed from concatenated sequences of 13 PCGs using Bayesian inference and the maximum likelihood method Posterior probability support from Bayesian inference and bootstrap support from maximum likelihood analysis are shown at each node, respectively. Scale bar indicates the number of substitutions per site. The Asiatic dhole (*Cuon alpinus*) was used as the outgroup to root the feliform tree.

## Additional Information and Declarations

### Data Availability

The following information was supplied regarding data availability: The raw data is available at NCBI GenBank: KX449332.

## References

[ref-1] Albert R (2001). Gene structure and gene flow in selected populations of spotted hyaena (Crocuta crocuta). Diss. Thesis.

[ref-2] Benton MJ, Donoghue PC (2007). Paleontological evidence to date the tree of life. Molecular Biology and Evolution.

[ref-3] Betley JN, Frith MC, Graber JH, Choo S, Deshler JO (2002). A ubiquitous and conserved signal for RNA localization in chordates. Current Biology.

[ref-4] Cosson L, Grassman Jr LL, Zubaid A, Vellayan S, Tillier A, Veron G (2006). Genetic diversity of captive binturongs (Arctictis binturong, Viverridae, Carnivora): implications for conservation. Journal of Zoology.

[ref-5] Darling AC, Mau B, Blattner FR, Perna NT (2004). Mauve: multiple alignment of conserved genomic sequence with rearrangements. Genome Research.

[ref-6] Darriba D, Taboada GL, Doallo R, Posada D (2012). jModeltest 2 more models, new heuristics and parallel computing. Nature Methods.

[ref-7] Drummond AJ, Rambaut A (2007). BEAST: Bayesian evolutionary analysis by sampling trees. BMC Evolutionary Biology.

[ref-8] Eizirik E, Murphy WJ, Koepfli KP, Johnson WE, Dragoo JW, Wayne RK, O’Brien SJ (2010). Pattern and timing of diversification of the mammalian order Carnivora inferred from multiple nuclear gene sequences. Molecular Phylogenetics and Evolution.

[ref-9] Flynn JJ, Finarelli JA, Zehr S, Hsu J, Nedball MA (2005). Molecular phylogeny of the Carnivora (Mammalia): assessing the impact of increased sampling on resolving enigmatic relationships. Systematic Biology.

[ref-10] Gaubert P, Cordeiro Estrela P (2006). Phylogenetic systematics and tempo of evolution of the Viverrinae (Mammalia, Carnivora, Viverridae) within feliformians: implications for faunal exchanges between Asia and Africa. Molecular Phylogenetics and Evolution.

[ref-11] Gaubert P, Veron G (2003). Exhaustive sample set among Viverridae reveals the sister-group of felids: the linsangs as a case of extreme morphological convergence within Feliformia. Proceedings of the Royal Society of London. Series B: Biological Sciences.

[ref-12] Gregory WK, Hellman H (1939). On the evolution and major classification of the civets (Viverridae) and allied fossil and recent Carnivora; a phylogenetic study of the skull and dentition. Proceedings of the American Philosophical Society.

[ref-13] Hunt RM (1991). Evolution of the aeluroid Carnivora: viverrid affinities of the Miocene carnivoran: Herpestides. American Museum Novitates.

[ref-14] Hunt Jr RM, Gittleman JL (1996). Biogeography of the order Carnivora. Carnivore behavior, ecology, and evolution.

[ref-15] Johnson WE, Eizirik E, Pecon-Slattery J, Murphy WJ, Antunes A, Teeling E, O’Brien SJ (2006). The late Miocene radiation of modern Felidae: a genetic assessment. Science.

[ref-16] Kearse M, Moir R, Wilson A, Stones-Havas S, Cheung M, Sturrock S, Buxton S, Cooper A, Markowitz S, Duran C, Thierer T, Ashton B, Mentjies P, Drummond A (2012). Geneious basic: an integrated and extendable desktop software platform for the organization and analysis of sequence data. Bioinformatics.

[ref-17] Kinnaird MF, O’Brien TG (2007). Ecology and conservation of Asian hornbills. Farmer of the forest. Chapter feeding ecology: how to survive on fruits.

[ref-18] Koepfli KP, Jenks SM, Eizirik E, Zahirpour T, Valkenburgh BV, Wayne RK, Molecular systematics of the Hyaenidae (2006). Relationships of a relictual lineage resolved by a molecular supermatrix. Molecular Phylogenetics and Evolution.

[ref-19] McKenna MC, Bell SK (1997). Classification of mammals above the species level.

[ref-20] Mohd Salleh F, Ramos-Madrigal J, Peñaloza F, Liu S, Mikkel-Holger SS, Riddhi PP, Martins R, Lenz D, Fickel J, Roos C, Shamsir MS, Azman MS, Lim BK, Stephen JR, Wilting A, Gilbert MTP (2017). An expanded mammal mitogenome dataset from Southeast Asia. GigaScience.

[ref-21] Morlo M, Miller ER, El-Barkooky AN (2007). Creodonta and Carnivora from Wadi Moghra, Egypt. Journal of Vertebrate Paleontology.

[ref-22] Nyakatura K, Bininda-Emonds OR (2012). Updating the evolutionary history of Carnivora (Mammalia): a new species-level super tree complete with divergence time estimates. BMC Biology.

[ref-23] Patou ML, Debruyne R, Jennings AP, Zubaid A, Rovie-Ryan JJ, Veron G (2008). Phylogenetic relationships of the Asian palm civets (Hemigalinae & Paradoxurinae, Viverridae, Carnivora). Molecular Phylogenetics and Evolution.

[ref-24] Pocock RI (1933). The rarer genera of oriental Viverridae. Journal of Zoology.

[ref-25] Raffles TS (1822). In Sir T. S. Raffles’s descriptive catalogue of a zoological collection made in Sumatra. The Transactions of the Linnean Society of London.

[ref-26] Rice P, Longden I, Bleasby A (2000). EMBOSS: the European molecular biology open software suite. Trends in Genetics.

[ref-27] Sambrook J, Fritsch EF, Maniatis T (1989). Molecular cloning: a laboratory manual.

[ref-28] Silvestro D, Michalak I (2012). raxmlGUI: a graphical front-end for RAxML. Organisms Diversity & Evolution.

[ref-29] Thompson JD, Higgins DG, Gibson TJ (1994). CLUSTAL W: improving the sensitivity of progressive multiple sequence alignment through sequence weighting, position-specific gap penalties and weight matrix choice. Nucleic Acids Research.

[ref-30] Veron G (2007). Phylogeny of the viverridae and ‘viverrid-like’ feliforms. Journal of Vertebrate Paleontology.

[ref-31] Veron G, Bonillo C, Hassanin A, Jennings AP (2017). Molecular systematics and biogeography of a Hemigalinae civets. European Journal of Taxonomy.

[ref-32] Veron G, Heard S (2000). Molecular systematics of the Asiatic Viverridae (Carnivora) inferred from mitochondrial Cytochrome b sequence analysis. Journal of Zoological Systematics and Evolutionary Research.

[ref-33] Willcox DH, Chutipong W, Gray TN, Cheyne S, Semiadi G, Rahman H, Coudrat CN, Jennings A, Ghimirey Y, Ross J, Fredriksson G (2016). Arctictis binturong. https://www.iucnredlist.org/species/41690/45217088.

[ref-34] Wozencraft WC, Wilson DE, Reeder DM (2005). Order Carnivora. Mammal species of the world—a taxonomic and geographic reference.

[ref-35] Yoder AD, Burns MM, Zehr S, Delefosse T, Veron G, Goodman SM, Flynn JJ (2003). Single origin of Malagasy Carnivora from an African ancestor. Nature.

[ref-36] Zhou Y, Wang SR, Ma JZ (2017). Comprehensive species set revealing the phylogeny and biogeography of Feliformia (Mammalia, Carnivora) based on mitochondrial DNA. PLOS ONE.

