# Supplementary material for: Characteristics of the complete mitochondrial genome of the monotypic genus Arctictis (Family: Viverridae) and its phylogenetic implications
Source: PeerJ. 2019 Nov 25;7:e8033. doi: 10.7717/peerj.8033 (PMC6882423; doi:10.7717/peerj.8033)
Supplement: Table S1 [file peerj-07-8033-s001.docx]

**Table S1**

**PCR Primer information used in this study.**

| **SET** | **PRIMER** | **Annealing temperature** | **PRODUCT SIZE** | **REFERENCE** |
| --- | --- | --- | --- | --- |
| **1.** | 12S-F | 47°C | 4.1kb | Kocher et al., 1989 |
|  | BINTU-IR7638 |  |  | **This study*** |
| **2.** | COX-1F | 51°C | 8.8kb | Folmer et al., 1994 |
|  | Mcb-R |  |  | Verma & Singh, 2003 |
| **3.** | Mcb-F | 48°C -51°C | 4.5kb | Verma & Singh, 2003 |
|  | 16S-R |  |  | Palumbi, 1996 |

**^*Sequence available on request^**

**List of References:**

Kocher TD, Thomas WK, Meyer A, Edwards SV, Paabo S, Villablanca FX and Wilson AC: Dynamics of mitochondrial DNA evolution in animals: Amplification and sequencing with conserved primers *Proc. Nati. Acad. Sci.* 1989.

Folmer O, Black M, Hoeh W, Lutz R and Vrijenhoek R: DNA primers for amplification of mitochondrial cytochrome c oxidase subunit I from diverse metazoan invertebrates. *Molecular Marine Biology and Biotechnology,* 1994.

Verma S and Singh L: Novel universal primers establish identity of enormous number of animal species for forensic application. *Molecular Ecology Notes*, 2003, DOI: 10.1046/j.1471-8286.2003.00340.x

Palumbi SR: Nucleic acids II: The polymerase chain reaction. In: Hillis DM, Moritz C, Mable BK (eds) Molecular systematics, 1996.
