# Supplementary material for: Characteristics of the complete mitochondrial genome of the monotypic genus Arctictis (Family: Viverridae) and its phylogenetic implications
Source: PeerJ. 2019 Nov 25;7:e8033. doi: 10.7717/peerj.8033 (PMC6882423; doi:10.7717/peerj.8033)
Supplement: Table S2 — The accession no. (KY117560) supplied for Binturong in Mohd Salleh et al. (2017) is in fact the accession for “Muntiacus muntjak mitochondrion, partial genome”. The Binturong sequence was obtained by contacting the authors directly. [file peerj-07-8033-s002.docx]

**Table S2**

**Mitogenomes used for comparative characterization and phylogenetic analysis, name of species, family and accession numbers**

| **Species** | **Family** | **Accession no.** | **Reference** |
| --- | --- | --- | --- |
| *Paguma larvata* | Viverridae | NC_029403.1 | Zhang et al., 2015 |
| *Viverricula indica* | Viverridae | NC_025296.1 | Weng et al., 2014 |
| *Genetta servalina* | Viverridae | NC_024568.1 | Hassanin, 2014 |
| *Arctictis binturong* | Viverridae | **#** | Salleh et al., 2017 |
| *Cynogale bennettii* | Viverridae | KY117544 | Salleh et al., 2017 |
| *Hyaena hyaena* | Hyaenidae | NC_020669.1 | Bon et al., 2011 |
| *Crocuta crocuta* | Hyaenidae | NC_020670.1 | Bon et al., 2011 |
| *Mungotictis decemlineata* | Eupleridae | NC_027828.1 | Hassanin, 2014 |
| *Herpestes javanicus* | Herpestidae | AY873843.1 | Penny and McLenachan, 2005 |
| *Nandinia binotata* | Nandiniidae | NC_024567.1 | Hassanin, 2014 |
| *Neofelis nebulosa* | Felidae | KU133958 | Tabasum et al., 2016 |
| *Panthera leo persica* | Felidae | KU234271 | Tabasum et al., 2016 |
| *Panthera leo leo* | Felidae | KF776494.1 | Wang, 2013 |
| *Leopardus colocolo* | Felidae | NC_028314.1 | Li et al., 2016 |
| *Catopuma badia* | Felidae | NC_028300.1 | Li et al., 2016 |
| *Lynx canadensis* | Felidae | NC_028313.1 | Li et al., 2016 |
| *Pardofelis marmorata* | Felidae | NC_028303.1 | Li et al., 2016 |
| *Caracal caracal* | Felidae | NC_028306.1 | Li et al., 2016 |
| *Leptailurus serval* | Felidae | NC_028316.1 | Li et al., 2016 |
| *Prionailurus viverrinus* | Felidae | NC_028305.1 | Li et al., 2016 |
| *Felis catus* | Felidae | NC_001700.1 | Lopez, 1995 |
| *Otocolobus manul* | Felidae | NC_028323.1 | Li et al., 2014 |
| *Prionodon pardicolor* | Prionodontidae | NC_024569.1 | Hassanin, 2014 |
| *Cuon alpinus* | Canidae | NC_013445.1 | Chen and Zhang 2009 |

# The Accession No. given in this reference does not belogs to *Arctictis binturong*, therefore, the sequence was taken from the authors on request.

**List of References:**

Zhang D, Xu L, Bu H, Wang D, Xu C and Wang R: The complete mitochondrial genome of themasked palm civet (Pagumalarvata, Mammalia,Carnivora. *Mitochondrial DNA*, 2015; DOI: 10.3109/19401736.2015.1079895

Weng HM, Wang L, Chan FT, Sun PY, Li KY and Ju YT: The complete mitochondrial genome of the small Indian civet,Viverricula indica taivana – the first complete representation ofthe genus Viverricula. *Mitochondrial DNA*, 2014; DOI: 10.3109/19401736.2014.958712

Hassanin A and Veron G: The complete mitochondrial genome of the Spotted Linsang, Prionodonpardicolor, the first representative from the family Prionodontidae(Mammalia, Carnivora). *Mitochondrial DNA*, 2014; DOI: 10.3109/19401736.2014.926482.

Hassanin A: The complete mitochondrial genome of the African palm civet,Nandiniabinotata, the only representative of the familyNandiniidae (Mammalia, Carnivora). *Mitochondrial DNA*, 2014.

Hassanin A: The complete mitochondrial genome of the Servaline Genet, Genettaservalina, the first representative from the family Viverridae (Mammalia,Carnivora). *Mitochondrial DNA*, 2014; DOI: 10.3109/19401736.2014.926479.

Bon C, Berthonaud V, Maksud F, Labadie K, Poulain J, Artiguenave F, Wincker P, Aury JM and Elalouf JM: Coprolites as a source of information on thegenome and diet of the cave hyena. *Proc. R. Soc. B* ,2012; doi:10.1098/rspb.2012.0358.

Li G, Davis BW, Eizirik E, Murphy WJ: Phylogenomic evidence for ancient hybridization in the genomes of living cats (Felidae). *Genome Res*, 2016; doi: 10.1101/gr.186668.114.

Salleh FM, Ramos-Madrigal J, Peñaloza F, Liu S, Sinding MHS, Patel RP, Martins R, Fickel DLJ, Roos C, Shamsir MS and Azman MS: An expanded mammal mitogenome dataset from Southeast Asia. *GigaScience*, 2017; doi: 10.1093/gigascience/gix053.

Tabasum W, Sreenivas A, Rai N, Thangaraj K and Gaur A: Complete mitochondrial genome sequence of Asiatic lion (*Pantheraleopersica*). *Mitochondrial DNA Part B: Resources*, 2016; doi.org/10.1080/23802359.2016.1214541.

Tabasum W, Sreenivas A, Bheemavarapu KK, Tirupathi RG and Gaur A Complete mitochondrial genome sequence of the Indian clouded leopard (*Neofelisnebulosa*). *Mitochondrial DNA Part B: Resources*, 2016.

**GenBank submission:**

Penny D, and McLenachan P, 2005;

Retrieved from https://www.ncbi.nlm.nih.gov/nuccore/AY873843.1

Chen L, and Zhang HH, 2009; Retrieved from https://www.ncbi.nlm.nih.gov/nuccore/NC_013445
