# Supplementary figures and images for: Characteristics of the complete mitochondrial genome of the monotypic genus Arctictis (Family: Viverridae) and its phylogenetic implications
Source: PeerJ. 2019 Nov 25;7:e8033. doi: 10.7717/peerj.8033 (PMC6882423; doi:10.7717/peerj.8033)

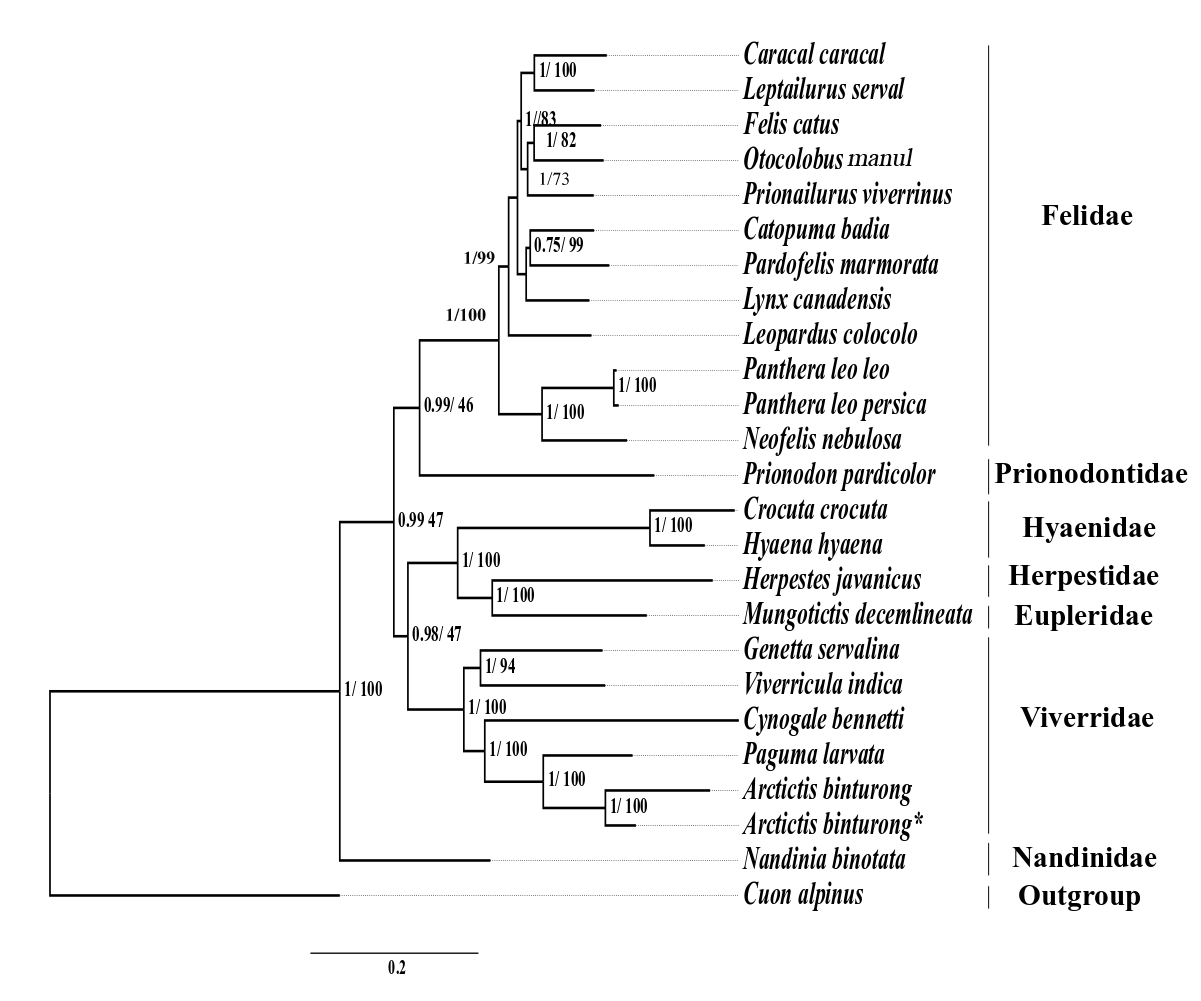

Supplement: Figure S1 — Posterior probability support from Bayesian inference and bootstrap support from maximum likelihood analysis are shown at each node, respectively. Scale bar indicates the number of substitutions per site. The Asiatic dhole (Cuon alpinus) was used as the outgroup to root the feliform tree. [file peerj-07-8033-s003.png]
